# Enhanced Dispersibility of Iron Oxide Nanoparticles Synthesized by Laser Pyrolysis with Isopropanol Vapors as Sensitizer

**DOI:** 10.3390/molecules31010163

**Published:** 2026-01-01

**Authors:** Iulia Ioana Lungu, Florian Dumitrache, Anca Criveanu, Lavinia Gavrila-Florescu, Ana-Maria Banici, Iuliana Morjan, Razvan-Mihai Dumitrache, Bogdan Vasile

**Affiliations:** 1 National Institute of Laser, Plasma and Radiation Physics, 409 Atomistilor Street, 077125 Magurele Ilfov, Romania; florian.dumitrache@inflpr.ro (F.D.); anca.criveanu@inflpr.ro (A.C.); lavinia.gavrila@inflpr.ro (L.G.-F.); ana.niculescu@inflpr.ro (A.-M.B.); iuliana.soare@inflpr.ro (I.M.); 2 Faculty of Physics, University of Bucharest, 077125 Bucharest-Magurele, Romania; razvan-mihai.dumitrache@s.unibuc.ro; 3 Department of Science and Engineering of Oxide Materials and Nanomaterials, University Politehnica of Bucharest, Gh. Polizu St. 1-7, 060042 Bucharest, Romania; bogdan.vasile@upb.ro

**Keywords:** iron oxide nanoparticles, laser pyrolysis

## Abstract

The present study investigates the synthesis and dispersibility process of iron oxide nanoparticles using laser pyrolysis with isopropanol vapors as a sensitizer agent. Similar to previous experiments (iron oxide nanoparticles synthesized by laser pyrolysis using ethylene as sensitizer gas), iron pentacarbonyl (Fe(CO)_5_) was employed as an iron precursor; however, instead of the classic ethylene, isopropanol was chosen as a sensitizer, which indicated beneficial features (especially enhanced dispersibility in water) in the as-synthesized nanoparticles. Structural and elemental analysis confirmed the size range of the nanoparticles (nanometric), with crystallite sizes under 10 nm. Both raw nanoparticles, as well as the oleic acid stabilized ones, exhibited excellent colloidal stability in both water and organic fluids (Toluene, Chloroform, and DMSO): around 100 nm hydrodynamic diameter and more than 40 mV for zeta potential. The study highlights the advantages of using isopropanol as a sensitizer in the production of high-purity iron oxide nanoparticles from laser pyrolysis, particles that showcase superior dispersibility and functionalization potential.

## 1. Introduction

Nanofluids and nanostructures based on magnetically structured crystalline nano-domains have various applications, such as high density magnetic information storage devices [1], drug delivery [2], as catalyst in the pharmaceutical industry [3], water pollution contaminated with organic pollutants through the catalytic process of the Fenton reaction [4], contrast element in diagnosis [5], and hyperthermia treatment [6].

The low dimensionality of nanomaterials leads to an unusually high ratio between the number of atoms on the surface layers compared to those in deep, volume layers, and this leads to significant physical–chemical changes [7,8]. In addition, there are noticeable differences between the chemical and physical behavior induced by surface atoms to the material if we are talking about a bulk material (crystalline or polycrystalline) or a particle or film with nanoscale dimensions [8]. Moreover, chemical and electrochemical properties are strongly influenced by the chemical composition and by the presence of dopants, impurities, or functional groups in the surface layers. An obvious example of the dramatic change in surface chemical properties is the chemical activity of gold (Au), which is known as inactive as a bulk material, but becomes an excellent catalyst in the colloidal/nanometric form [9]. Magnetism is also influenced when comparing the same material in bulk or at the nanoscale. Such elements with unnoticeable magnetism, such as platinum (Pt), may exhibit magnetic susceptibility as the size decreases to clusters of dozens of atoms [10]. This paper will focus on specific experimental conditions for obtaining iron oxide nanoparticles and the effect of these parameters on the final properties of the material, including its dispersion in different fluids. The nanoparticles (NPs) were synthesized through laser pyrolysis. A focused laser beam (CO_2_ laser in quasi-continuum regime) interacts with at least one of the gaseous species in the precursor mixture, resulting in a transfer of light energy into thermal energy that initiates the decomposition processes and the generation of condensable species [11]. Laser pyrolysis is a synthesis process from the gas phase, in an open environment, and with precursors in a stationary flow; the reaction time, determined by the residence time in the flame, is between tens of microseconds and several milliseconds. The reaction area is uniform, and the thermal pump source is a constant one in time and space. Reactive precursors must also meet certain requirements; they must mainly be in the form of gas or possibly vapor from a rather volatile liquid. The precursor most commonly used in iron-based pyrolysis synthesis is iron pentacarbonyl—Fe(CO)_5_. It has a significant volatility (38 mbar at 25 °C according to [12]), but has no absorption over the laser wavelength. Studies on the sequential decarbonylation by laser pyrolysis of Fe(CO)_5_ [13,14,15,16] have revealed that carbonyl ligands are rapidly removed (the first bonding energy is 41.5 kcal/mol), with the formation of metallic iron and CO:

Fe(CO)_5_ = Fe(CO)_x_ + (5 − x) CO, x = 0–4. (1)

Thus, the final product of rapid decarbonylation should be CO and iron particles, but it should be taken into account that the degree of decomposition obviously depends on the reaction temperature, which in turn will depend on the irradiation parameters. Moreover, the decomposition of Fe(CO)_5_ in the pyrolytic flame and the formation of clusters in the presence of excess oxygen favor the highest iron ionization, Fe^3+^. As mentioned earlier, magnetic NPs are used in important and various applications, and their synthesis techniques are numerous. Special attention should be paid to the functionality, coating, or activation of the surface of these nanomaterials or nanocomposites. Depending on the desired application, one can choose the type of magnetic nanomaterial, the technique for obtaining it, or the method of operation/coating, which can be added even in the synthesis process (one-step methods of synthesis of functional or hybrid magnetic nanomaterials). Magnetic NPs are widely used in combination with other nanoparticles, nanostructures, molecular compounds, or macromolecules, forming hybrid nanomaterials. An important role of magnetic nanoparticles is related to the caloric effect induced by nanoparticles in interaction with a radiofrequency electromagnetic field (Neel effect). The phenomenon of hyperthermia applies to magnetic nanomaterials coated in polymers or biocompatible molecules in the case of tumor treatment [17].

A wide range of applications of magnetic nanoparticles requires the obtaining of magnetorheological fluids. These are materials with absolutely surprising properties; under the action of a magnetic field, magnetic fluids transform from liquid consistency (Newtonian liquid in the absence of a magnetic field) into quasi-solid (increased shear viscosity induced by the interaction of magnetic nanoparticles and their ordering as magnetic dipoles), and this phenomenon is rapid and reversible [18].

Certainly, magnetic fluids based on superparamagnetic materials or possibly soft magnets are superior to those based on particles with residual magnetization because they are easy to magnetize and then demagnetize, thus the rheological properties are controlled by low-intensity magnetic fields. Magnetic fluids using micrometric magnetic particles have abrasion potential and also the tendency to sediment due to their higher net density compared to the density of the base fluid, for which reason magnetic fluids based on nanoparticles and possibly superparamagnetic NPs are all the more suitable. Essential for magnetorheological applications is the dispersion of nanomaterials in the base fluid and for them to be properly functionalized (electrostatic or steric).

Laser pyrolysis with the use of Fe(CO)_5_ requires another species to absorb laser energy—a sensitizer gas. An example of such a gas is C_2_H_4_—ethylene. Near the frequency of the laser radiation: 944 cm^−1^, there are two vibration levels with two fundamental frequencies of 949.3 cm^−1^ and 940.6 cm^−1^, so ethylene has a significant absorption, characterized by the absorption coefficient of 1.7 × 10^−3^ torr^−1^ cm^−1^ [19]. Another species with weaker absorption, but used for the same purpose, is isopropanol vapors; literature indicates a value of 0.003 m/N at 10.3 Torr [20]. The use of this weaker sensitizer comes with the advantage that after the laser–molecule interaction, the energy transfer is lower as compared to ethylene. The excitation of this on the resonant level, then the process of thermalization of it after intermolecular collisions, in particular, is attained without heavily affecting the decomposition of the sensitizer. Therefore, the carbon (C) content of iron oxide samples obtained by laser pyrolysis using isopropanol as a sensitizer is lower than when ethylene is used.

A first study presented here focuses on the selection of the type of sensitizer and weight of molecular oxygen in the oxidizing mixture (AR + O_2_), the pressure, and the value of the radiating laser power, in which high-purity nanoscale iron oxides obtained have been optimized in previous studies. The pressure is maintained constant, and the primary power regime is set so that the temperature in the flame is between 500 and 700 °C. The next step involved the optimization of preparing a stabilized suspension based on the magnetic NPs synthesized in the previous steps.

Oleic acid (OA), a mono-unsaturated omega-9 fatty acid, has amphiphilic properties with a COOH- polar bond, and it has been proven to be an excellent surfactant to modify the surface of magnetite nanoparticles due to the high affinity to its surface [21]. Scientific studies confirm the chemical bonding (chemisorption) between the carboxyl group of OA and iron ions on magnetite/maghemite nanoparticle surfaces. Oleic acid forms a carboxylate structure, often binding in a bidentate geometry with surface iron atoms, ensuring stable anchorage [22]. OA molecules act as a capping agent at the nanoparticle surfaces, generating a steric stabilization in organic base fluids due to their hydrophobic tail [23]. Additionally, in order to stabilize maghemite/magnetite nanoparticles in aqueous base fluids, a two-step coating process is employed. First, a hydrophobic layer of OA is chemically adsorbed directly onto the iron oxide surface. Subsequently, a second layer is physically attached to the first, with its hydrocarbon tails interacting with the initial OA layer, leaving its polar carboxylic groups facing outward toward the fluid interface [24]. Furthermore, OA is an extremely widespread natural fatty acid and a natural and essential component of living organisms, which means its biocompatibility is first-rate. Its natural presence in living structures such as the lipid shell of cell membranes [25,26] suggests that the body easily recognizes and integrates it, generally without activating an immune or toxic response. Some studies have demonstrated exceptional stabilization, due to the formation of strong chemical bonds between the carboxylic acid and the amorphous iron oxide nanoparticles [27,28,29]. Due to magnetic dipole interactions, the OA layer disperses the NPs, thereby producing highly uniform and monodisperse particles and preventing them from forming large clusters [30].

## 2. Results and Discussion

### 2.1. Nanoparticle Characterization

Iron oxide nanoparticles were synthesized using the set-up presented in the Materials and Methods section. When a carrier gas passes through a bubbler vessel, containing volatile liquids (IZP or Fe(CO)_5_), the expected value of the vapor flow is given by the following equation:

(2) $$\Phi_{vapour}=\Phi_{carrier}\cdot \frac{p_{vapour}^{sat}}{p-p_{vapour}^{sat}}$$

where $p_{vapour}^{sat}$ is the pressure of the saturated vapours and p is the pressure of the reactive chamber.

For example, in the case of an Ar flow of 50 Standard Cubic Centimeters per Minute (sccm) that enters the bubbler containing Fe(CO)_5_ in liquid form, at an ambient temperature of 25 °C, the estimated flow will be 7.35 sccm, determined by the equation presented above [31].

Iron pentacarbonyl pressure vapors were determined by the Antoine Equation using the parameters provided by webbook.nist.gov (accessed on 10 October 2025). The same method can be used to calculate the flow of isopropanol vapors.

**Figure 1:**
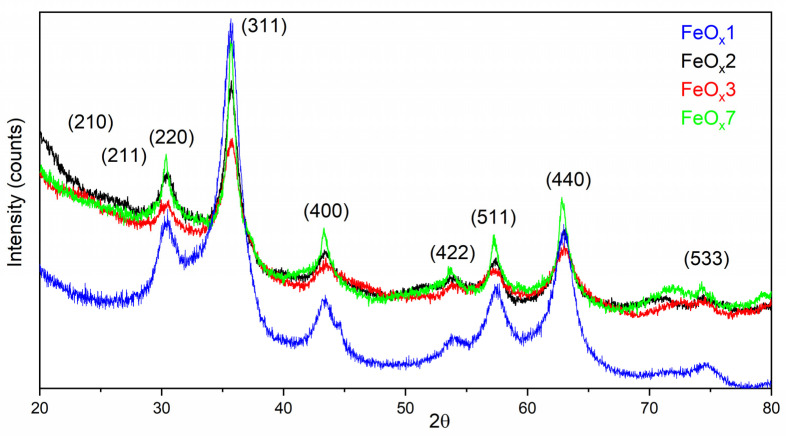
X-ray diffractograms for powders obtained with isopropanol as a sensitizer.

For the samples in the experimental study of the synthesis of iron oxide NPs with isopropanol as a sensitizer, the diffractograms are shown in Figure 1. To improve visibility, the analyzed samples were chosen according to their total flow: highest value—FeO_x_3, lowest—FeO_x_7, middle—FeO_x_1, and FeO_x_2, because it exhibited the best results throughout our experiments. The analyzed material is a nanometric one, the peaks are broad and suitably fitted with a pseudo-Voigt function, and the full width half maximum (FWHM) was evaluated from this function. The mean crystallite size was measured using the Scherrer Equation (neglecting the microstrain influence) applied on the (440) peak (the second one as intensity), an isolated peak, as compared to the (311) peak that can be found at ~35.6°. Based on the position and half-length width of the peak (440), located at approximately 2θ = 63.0°, the order (crystallite) size of the nanocrystalline material under analysis is determined (see Table 1). Additionally, for the FeO_x_ 1, 2, 3, and 7 samples, a combined evaluation of the mean crystal size and microstrain was attained using the Williamson–Hall technique. The FWHM (β) of the peaks from (311), (400), (511), and (440) were evaluated, the linear fitting of the dependence β·cos(θ) = f(4·sin(θ)) was plotted, then D_mean_ and **ϵ** for each sample were determined (Table 1, last two columns). We observe that the difference compared to the value calculated by neglecting the contribution of microstrain is minimal. It is observed that there is a downward trend in microstrain as the order size (D_mean_) increases. The evaluation of the D_mean_ values using the Williamson–Hall technique appears to be more precise for samples with larger nanoparticles (all the points are closer to the linear fitting curve), probably because the FWHM value is better evaluated due to a higher signal-to-noise ratio.

There is no clear evidence of the specific peaks for ordered maghemite (210) and (211) for the examined samples, making the distinction between magnetite and disordered maghemite problematic since their crystalline structure is quite similar—cubic crystallization, space group P4132(123). By evaluating the lattice parameters for sample FeO_x_2 based on the diffraction peaks (440) and (311), the values of the lattice parameter are a = 8.3519 Å and a = 8.3515 Å. These measurements are consistent with the typical values for magnetite (~8.39 Å) and maghemite (~8.35 Å) [32], indicating the sample is likely pure maghemite or maghemite with low magnetite content.

Shortly, the residence time is the time spent by the reactive gases in the reaction zone, and strongly influences the temperature in the pyrolytic flame, and subsequently the size of the freshly nucleated particles (the longer they stay in the reaction zone, the higher their growth). An in-depth overview of how the residence time is calculated and how it affects the final particles, as presented in a previous study [33]. The crystallite size increases with increasing residence time; thus, only by controlling the total flow and maintaining a constant proportion between the gases in the central mixture, the crystallite size can be controlled, implicitly their superparamagnetism characteristic at room or lower temperature, as well as the magnetic susceptibility of the studied materials (powder or suspension) [34].

The elemental composition assessments can be observed in Table 2. It can be seen that the oxygen content exceeds the phase with the highest ionization state of Fe: Fe^3+^. The reason for this unusual behavior is the surface presence of oxygen, through additionally adsorbed molecular oxygen: the presence of C-O-O-H, Fe-O-H, and C-O-H functional groups.

**Figure 2:**
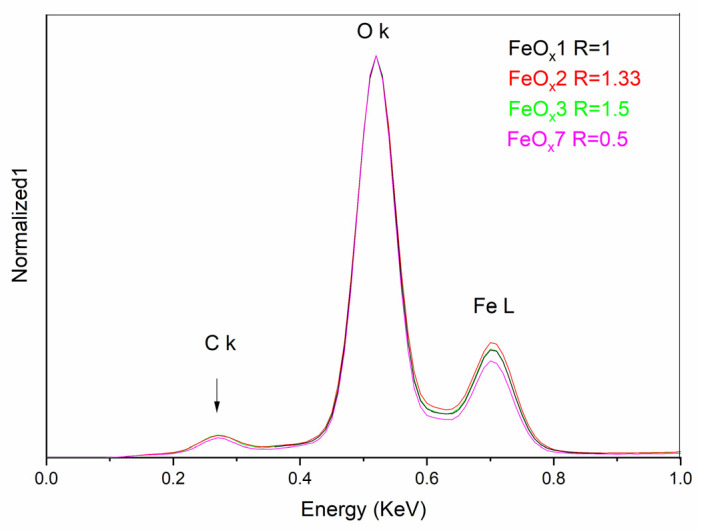
EDS spectra for samples synthesized with isopropanol as a sensitizer.

Figure 2 shows the active zone for the lighter elements C _K_, O _K_, and Fe _L_ that can be identified. For easier differentiation, the sample FeO_x_1 is considered as a reference, its total reactive flow being weighted with R = 1, meaning 136 sccm, the rest of the samples referring to this value. The energy-dispersive X-ray spectroscopy (EDX) evaluation in the approximations used by the apparatus (ZAF—standardless) tends to underestimate the percentage contribution of heavier elements (Fe) relative to the proportion of lighter ones (C and O) [35,36]. The amount of carbon present is significantly lower compared to experiments that used ethylene as a sensitizer [37] and comparable with the experiments that used ethanol [38]. This low carbon content can be attributed to the pyrolytic decomposition of isopropanol. The diminished presence of carbon in the composition of iron oxide nanoparticles, compared to methods using ethylene, suggests a reduced superficial coating with amorphous carbons or polyaromatic residues. This leaves the surface of the nanoparticles dominated by the polar iron oxide groups, thereby creating the premises for a hydrophilic surface and a favorable chemosorption of OA via its carboxylic group.

X-ray photoelectron spectroscopy (XPS) analysis was performed on the FeO_x_2 sample in order to evaluate the surface composition and the iron bonding/oxidation states. The survey spectrum revealed the presence of Fe, O, and C at 27.8, 53.8, and 18.4 at.% respectively. The higher carbon content observed by XPS compared to EDX indicates its preferential surface enrichment.

**Figure 3:**
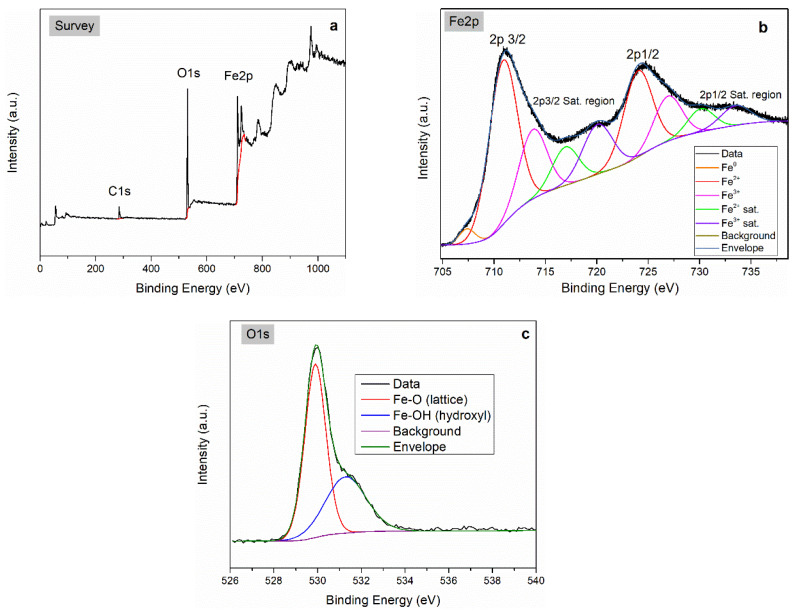
XPS analysis for sample FeO_x_2: (**a**)—XPS survey, (**b**)—High resolution at Fe 2p active zone, and (**c**)—High resolution at O 1s active zone.

The high-resolution Fe 2p region (705–717 eV) shows a main Fe 2p_3_/_2_ peak centered at ~711.0 eV, indicating the dominant presence of Fe^3+^ species relative to Fe^2+^ (~709.4 eV) or metallic Fe^0^ (~707.1 eV). This assignment is further supported by the characteristic Fe^3+^ satellite feature at ~720.0 eV and by the deconvolution of both the Fe 2p_3_/_2_ and 2p_1_/_2_ components (Figure 3b, right). The presence of Fe^0^ is also clearly identified, as highlighted by the orange fitted component. For clarity, the most relevant fitted components (Fe^0^—orange, Fe^3+^ main peak—red, Fe^3+^ satellite—violet, background—dark green) are displayed with thicker lines on the right side of Figure 3 [39].

The O 1s spectrum of FeO_x_2 (Figure 3c) is composed of two main components centered at 529.9 eV and 531.3 eV, which are assigned to lattice oxygen in Fe–O bonds and surface hydroxyl groups (Fe–OH), respectively. Minor contributions from C–O species at higher binding energies cannot be fully excluded. The high relative proportion of Fe–OH groups (~39 at.% O) suggests that these nanoparticles present a highly hydroxylated surface, which is favorable for stabilization in polar media or in organic solvents using amphiphilic ligands (e.g., oleic acid).

**Figure 4:**
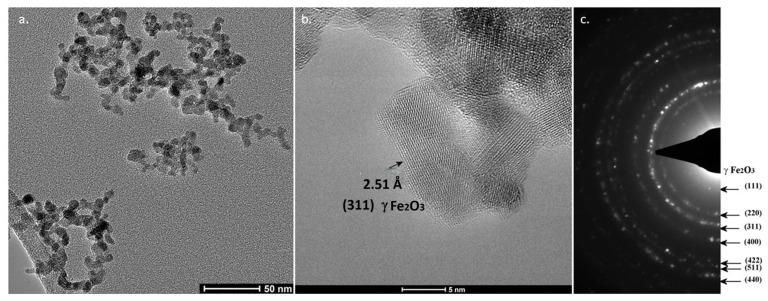
TEM micrograph from sample FeO_x_2 at different magnifications (**a**,**b**) and SAED analysis (**c**).

In Figure 4a,b, TEM images at two magnifications of the as-synthesized powder are presented for sample FeO_x_2. Most of the particles have a uniform spherical shape and are self-assembled in chain-like structures (Figure 4a). A particle statistic made from Figure 4a using ImageJ software 1.46r revealed a monodisperse size distribution centered at 5.9 nm, very close to X-ray Diffraction (XRD) results regarding mean crystalline dimension. Taking this into consideration, we can conclude that the nanoparticles have a monocrystalline domain, for the most part. At higher TEM magnification (Figure 4b), rather round surfaces can be observed, and the distance identified between two adjacent atomic planes was 2.5 Å, attributed to (311) γ-Fe_2_O_3_ crystalline phase. According to the SAED analysis (c), associated rings ascribable only to γ-Fe_2_O_3_ and/or Fe_3_O_4_ [(2 2 0), (3 1 1), (4 0 0), (5 1 1), (4 4 0)] could be identified. The observation that the electron diffraction rings appear broad with broad spots is a characteristic feature directly attributed to the nanometric structure of the iron oxide phase. This broadening is a consequence of the extremely small crystallite size of the material, typically in the range of a few to ten nanometers in accordance with the Scherrer principle (which applies to both X-ray and electron diffraction); as the crystallite size decreases, the width of the diffraction peaks (or rings in a crystalline pattern) increases.

Dynamic light scattering (DLS) is a non-invasive technique that can study the aggregation of particles in a suspension in a fast, accurate, and reproducible manner without using large quantities of the sample analyzed [40]. The stability guidelines for zeta potential values state that a suspension that has ‘good stability’ has zeta potential values that range between ±40 and ±60 mV [41]. DLS analysis showed that the majority of the NPs present a high degree of stability in water-based suspension, having a zeta potential of above 30 mV, making them colloidally stable [42]

### 2.2. Stabilization with Oleic Acid

As previously mentioned, iron oxide nanoparticles can be used in a variety of applications, especially in the biomedical field for drug delivery [43,44,45], magnetic imaging [46,47,48], and hyperthermia [49,50,51]. However, in order for these NPs to be suitable for said applications, specific requirements need to be met, including, but not limited to, biocompatibility, colloidal stability, specific size, shape, and magnetic behavior, depending on the requirements of each application.

Since iron oxide nanoparticles have a high tendency of agglomeration, it is critical that the formation of aggregates in suspension is prevented for biomedical applications because they are directly linked to their biodistribution—size (either of the individual or the aggregates) will directly affect the blood stream clearance of the nanoparticles. Several studies have reported that correlate the size range of nanoparticles and clearance mechanisms exhibited by the body. For example, nanoparticles larger than 200 nm have a faster removal rate due to the phagocytosis generated by the mononuclear phagocyte system. Particles higher than 100 nm generally accumulate in the liver and spleen and are eliminated by macrophages, whereas particles smaller than 10 nm lead to renal clearance. Nanoparticles that have a size range from 10–100 nm have been reported as desired candidates for clinical applications. Moreover, for tumor tissue retention and accumulation, the golden standard in regards to nanoparticle/aggregate size might be around 50 nm [52,53,54].

Due to the obtained results, FeO_x_2 was further tested regarding its stabilization with OA in organic fluids (Toluene, Chloroform, and Dimethyl sulfoxide (DMSO)). The following protocol was used: the solutions contained equal concentrations of NPs and OA (2 g/L) in a final volume of 50 mL of Toluene, Chloroform, and DMSO. The treatment was as follows: ultrasound bath starting from 25 °C and going up to 70 °C for approximately 90 min at 59 kHz. After 90 min, the bath was stopped, and the suspension was left to cool down slowly for another two hours. For a better understanding of the stabilization, a DLS analysis was obtained immediately after the two-hour cooling period, and 20 h after.

Dynamic light scattering analysis is an excellent tool that can be used to analyze particles in suspensions and determine their hydrodynamic diameter, the polydispersity index, as well as the stability of the suspension. Typically, PDI values between 0.1 and 0.7 are attributed to near monodisperse suspensions [40,55]. The results from Table 3 suggest excellent stability, and the NPs’ agglomerate dimension is extremely reduced. The values of the polydispersity index indicate an exceptional suspension of the NPs with a distribution close to monodisperse [56]. The results can be explained by the binding mechanisms of OA onto the surface of iron oxide nanoparticles. Briefly, the carboxylate headgroups chemically absorb onto the surface of the NPs, while the hydrocarbon tails form a hydrophobic shell, which generates steric stabilization. The colloidal stability of such suspensions is dependent on the interaction between the OA tail and the solvent used. A clear example is the use of Toluene, a nonpolar solvent with a strong effect on the OA chains. Due to these interactions, a thick steric barrier can be formed around the NPs, which explains the DLS results: the inconsequential difference in size between 2 h and 20 h could be explained by steric repulsion from particle–particle contact, also resulting in a low PDI. A similar effect was observed for Chloroform, however, with slightly higher values because it is a weakly polar solvent. Lastly, DMSO, a highly polar solvent, though efficient, displayed higher DLS values with a slight increase from 2 h to 20 h. This can be explained by the weak tail–solvent interaction, which leads to loss of the steric barrier around the NPs and subsequently to aggregation. 

**Figure 5:**
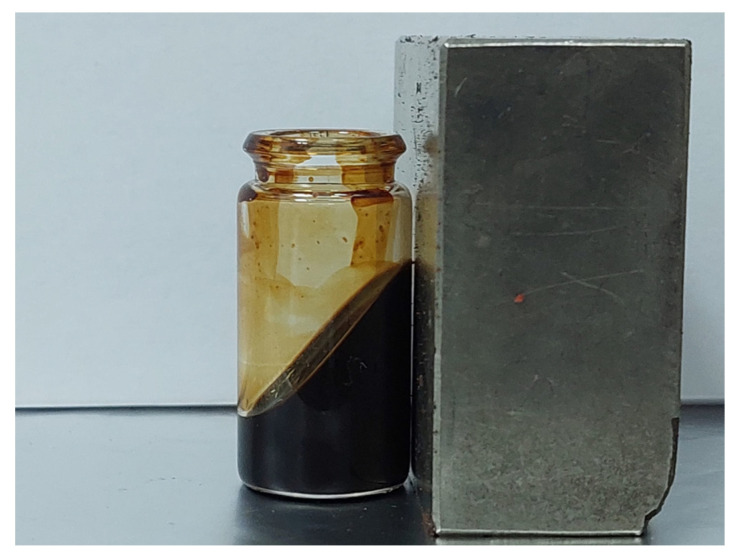
NPs suspension in organic fluid (chloroform) with high magnetic response.

Figure 5 presents the magnetic behavior of a concentrated suspension of iron oxide NPs, FeO_x_2, and oleic acid in chloroform, where the concentrations were 8 g/L NPs + 8 g/L OA.

## 3. Materials and Methods

### 3.1. Synthesis of Iron Oxide Nanoparticles

**Figure 6:**
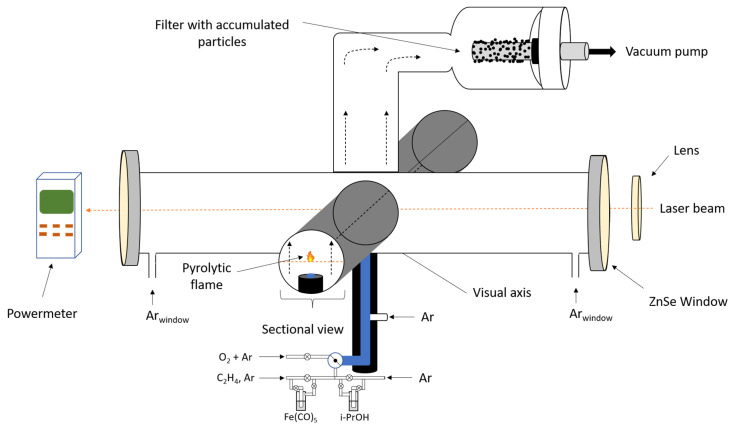
Experimental installation: magnetic iron oxide nanoparticles.

Figure 6 is a schematic representation of the synthesis of iron oxide nanoparticles through laser pyrolysis using isopropanol (iPrOH, Chimreactiv S.R.L., Bucharest, Romania) as a sensitizer. A CO_2_ laser (Puri Laser Technology CO, Nantong, China) in a quasi-continuum regime is used, which operates at a λ = 10.59 µm, the fundamental frequency in the emission module being 10P20. The laser beam is focused and enters the reaction chamber through ZnSe optical transparent windows that have an anti-reflective layer calibrated for the laser’s wavelength. The pressure inside the reaction chamber is kept constant by an evacuation system equipped with a vacuum pump controlled by an electromagnetic valve based on flow. Moreover, the gas flow is controlled through flow regulators. Iron pentacarbonyl (Fe(CO)_5_) (Merk KGaA, Darmstadt, Germany) is used as a Fe precursor, isopropanol as a sensitizer, and Ar (Messer SE & Co. KGaA, Bad Soden, Germany) flows are used to maintain the precursor gas and the freshly nucleated particles on the flow axis, as well as keeping the windows free from impurity. For security reasons, Fe(CO)_5_ is stored at low temperatures (−20 °C). The main vessel is introduced in the glove box, and the injection of the liquid in the bubbler is attained inside it in a nitrogen preponderant atmosphere, therefore keeping the liquid at low temperatures during manipulation. After isolating the bubbler, the atmosphere was refreshed with nitrogen so that no residual vapors are present when taking the primary vessel out for storage.

The following samples, FeO_x_1 through FeO_x_7, were synthesized using a focal lens (F = 25 cm) with a reaction zone of 26.5 cm, which corresponds to a circular focal point of φ = 1.5 mm. The pressure and the laser power were kept constant during the experiments at 300 mbar and 305 W, respectively, while the flow for the reactive mixes varied throughout the experiments (see Table 4).

### 3.2. Morpho-Structural Characterization

The above-mentioned samples have been characterized using the DLS (Nanoparticle Analyzer SZ-100V2, Horiba, Kyoto, Japan) technique in order to attain the hydrodynamic diameter, the polydispersity index, and the zeta potential. The pH in the suspension was also measured. The suspensions were obtained by adding the appropriate quantity for 0.5 g/L of powder in 50 mL of distilled water. The treatment used was 30 min in an ultrasound bath at 59 kHz, and 5 min under a sonotrode, at 500 W. For a better understanding of the electrophoretic mobility, the samples were also diluted down to a 0.1 g/L concentration (see Table 5).

The crystallinity of raw nanopowders was evaluated by a PANalytical X’Pert^3^ Powder X-ray diffractometer (XRD) (Malvern, Panalytical Ltd., Malvern, UK) equipped with a θ-θ vertical goniometer and using a Cu K_α_ radiation source (wavelength of 1.5418 Å).

Energy-dispersive X-ray spectroscopy (EDX) measurements for elemental analysis were performed within a FEI Quanta Inspect F50 S scanning electron microscope (SEM, Thermo Fisher Scientific Inc., Waltham, MA, USA) at 10 kV accelerating voltage, equipped with an energy-dispersive X-ray analysis (EDAX) facility (ELEMENT Silicon Drift Detector). X-ray photoelectron spectroscopy (XPS) analysis was attained using an ESCALAB Xi+ (Thermos Scientific Surface Analysis, Waltham, MA, USA) apparatus that employed an Al Kα radiation source (hν = 1486.2 eV). Bright-field Transmission electron microscopy (TEM) micrographs were obtained with the use of a Titan Themis transmission electron microscope (Thermo Fisher Scientific, Waltham, MA, USA), which operated at a voltage of 200 kV in transmission mode.

## 4. Conclusions

This study focused on the dispersibility and carbon content of iron oxide nanoparticles resulting from laser pyrolysis when isopropanol was used as a sensitizer. For a better comparison and deeper understanding, seven samples were synthesized varying the gas flows, but maintaining the pressure and power regime constant. The elemental and structural characterizations confirmed the nature of the particles, and the DLS analysis indicated excellent stability of the samples in distilled water. Further, a reference sample was tested regarding its stability in organic fluids, with the addition of oleic acid. The results displayed extremely reduced NP agglomerations with exceptional stability, even after 20 h. Moreover, this protocol did not hinder the magnetic behavior of the NPs.

## Figures and Tables

**Table 1:** Crystallite size and diffraction peak position (440).

Sample	D_mean_ (nm)	2θ-440	D_mean_ (nm)	ε
FeO_x_1	5.00	62.99	5.32	0.0018
FeO_x_2	5.95	62.97	6.22	0.0012
FeO_x_3	4.25	63.05	4.27	0.0027
FeO_x_7	9.08	62.94	9.93	0.0010

**Table 2:** Elemental composition of iron oxide NPs extracted from Electron Dispersion Spectroscopy (EDS) spectra.

Sample	C	O	Fe	X from Fe_2_O_3+x_
FeO_x_1	1.7	62.4	35.9	0.48
FeO_x_2	1.7	61.3	37.0	0.31
FeO_x_3	1.9	62.0	36.1	0.43
FeO_x_7	1.8	63.6	34.6	0.67

**Table 3:** DLS analysis of the obtained suspensions (the results presented are the average of three consecutive analyses).

Sample	Z-Average (nm) 2 h	PDI 2 h	Z-Average (nm) 20 h	PDI 20 h
FeO_x_2_OA_Toluene	39.4	0.153	39.7	0.168
FeO_x_2_OA_Chloroform	55.9	0.224	55.5	0.222
FeO_x_2_OA_DMSO	132.8	0.211	138.9	0.185
NPs + OA + C	**DLS 5 min**		**DLS 1 h**	
	**Z-average**	**PDI**	**Z-average**	**PDI**
C_2_OA	59.6 nm	0.276	61.9 nm	0.314
C_2_OA_x4	56.0 nm	0.255	54.7 nm	0.220
C—chloroform, 2—FeO_x_2, OA—oleic acid, ×4—concentration ×4				

**Table 4:** Main experimental parameters.

Sample	D_central_ (sccm)			D_ext_ (sccm)	T_f_ (°C)	τ
	D_Ar/Fe_	D_Ar/iPrOH_	D_O2_			(msec)
FeO_x_1	50/7.25	50/8.59	20	2000	515	0.2805
FeO_x_2	66/9.57	66/11.34	26	2640	505	0.2156
FeO_x_3	75/10.84	75/12.89	30	3000	500	0.1906
FeO_x_4	33/4.78	33/5.67	13.2	1333	600	0.3835
FeO_x_5	25/3.62	25/4.29	10	1000	620	0.4950
FeO_x_6	12.5/1.81	12.5/2.14	5	500	615	0.9958
FeO_x_7	6.25/0.90	6.25/1.07	2.5	250	610	2.0035

**Table 5:** DLS measurements.

Sample	Z-Average (nm)	PDI	Zeta Potential (mV)	pH
FeO_x_1	107.4	0.425	58.4	4.79
FeO_x_2	95.9	0.373	49.3	4.71
FeO_x_3	112.2	0.371	46.6	3.45
FeO_x_4	72.7	0.292	33.5	3.72
FeO_x_5	114.4	0.446	58.7	3.82
FeO_x_6	125.5	0.414	58.1	4.65
FeO_x_7	202.1	0.479	13.77	4.90

## Data Availability

The original contributions presented in this study are included in the article. Further inquiries can be directed to the corresponding author.
